# Unravelling the Acute, Chronic and Steroid-Refractory Management of High-Grade Neurological Immune-Related Adverse Events: A Call to Action

**DOI:** 10.3390/brainsci14080764

**Published:** 2024-07-29

**Authors:** Antonio Malvaso, Pierpaolo Giglio, Luca Diamanti, Matteo Gastaldi, Elisa Vegezzi, Andrea Pace, Paola Bini, Enrico Marchioni

**Affiliations:** 1 Department of Brain and Behavioral Sciences, University of Pavia, 27100 Pavia, Italy; antonio.malvaso01@universitadipavia.it (A.M.); pierpaolo.giglio01@universitadipavia.it (P.G.); 2 Neuroimmunology Research Unit, IRCCS Mondino Foundation—National Neurological Institute, 27100 Pavia, Italy; matteo.gastaldi@mondino.it; 3 Neuroncology Unit, IRCCS Mondino Foundation—National Neurological Institute, 27100 Pavia, Italy; luca.diamanti@mondino.it (L.D.); elisa.vegezzi@mondino.it (E.V.); paola.bini@mondino.it (P.B.); 4 IRCCS Regina Elena, Istituto Nazionale Tumori, 00144 Rome, Italy; andrea.pace@ifo.it

**Keywords:** neurological toxicities treatment, high-grade n-irAEs, immune checkpoint inhibitors, personalized treatment, steroid-resistant n-irAEs

## Abstract

Rare side effects of immune-checkpoint inhibitors (ICIs) are known as neurological immune-related adverse events (n-irAEs). Typically, n-irAEs affect the peripheral nervous system, primarily presenting as myositis, polyradiculoneuropathy, or cranial neuropathy. Less commonly, they impact the central nervous system, resulting in encephalitis, meningitis, or myelitis. High-grade n-irAEs managing and recognizing remains challenging, considering the risk of mortality and long-term disability. To date, strong scientific data are lacking to support the management of high-grade clinical forms. We performed a systematic literature search, selecting all articles describing high-grade steroid-resistance n-irAEs. and we reported them in a practical review. Specifically, current recommendations advise stopping ICI use and beginning corticosteroid treatment. Our findings highlighted that in steroid-resistant n-irAEs, it should be recommended to quickly escalate to plasma exchange (PLEX) and/or intravenously immunoglobulins (IVIg), usually in association with other immunosuppressants. Furthermore, newer evidence supports the use of drugs that may specifically block inflammation without reducing the anti-tumour effect of ICIs. In this practical review, we provide new evidence regarding the therapeutic approach of high-grade n-irAEs, particularly in steroid-resistant cases. We would also stress the importance of informing the scientific community of the discrepancy between current guidelines and clinical evidence in these rare forms of pathology.

## 1. Introduction

One significant advancement in the treatment of cancer has been the use of ICIs [1]. ICIs are directed against molecules including PD-1, its ligand PDL-1 and CTLA-4 [2]. It is well known that they can induce rare immune-related neurological adverse events (n-irAEs) that are frequently severe and associated with a significant risk of death and permanent disability. N-irAEs may impact the CNS, resulting in encephalitis, meningitis, or myelitis, whereas typically they affect the PNS, primarily presenting as myositis, polyradiculoneuropathy, or cranial neuropathy [3,4]. When compared to other ICI-related toxicities, n-irAEs occur infrequently—about 1% of the time [5], and their incidence is rising in tandem with the expansion of ICI oncological indications [5]. Because of their impact on any possible area of the nervous system, many phenotypes may coexist in a single patient, presenting with a wide range of clinical manifestations. 

According to a recent comprehensive systematic review of the literature [5], neuromuscular manifestations occur three times more frequently than the involvement of the CNS. Interesting correlations have been recently observed between the types of ICIs, the features of the cancer, and the clinical presentation [5]. The Common Terminology Criteria for Adverse Events (CTCAEs) grades the severity of n-irAEs, according to a scale ranging from 1.0 (mild: symptoms interfering with everyday activity or instrumental life) to 5.0 (death due to the symptoms) [6]. Most n-irAEs are usually mild (e.g., non-disabling headache, dizziness, or peripheral neuropathy; CTCAE < III) [7]. Severe or high-grade n-irAEs (CTCAE > II) are less common than other toxicities, although they nonetheless affect a significant percentage of treated individuals (1–3%) and can be life-threatening, leading to a high degree of disability [8,9,10,11]. Therefore, it is noteworthy to consider prompt treatment to reduce worsening outcomes and mortality risk in these cases. Steroids aer considered the first-line treatment of many n-irAEs, although rare patients are refractory to this management strategy and require additional immunosuppressants or IVIg/PLEX as a second-line treatment. Since cases of corticosteroid-refractory n-irAEs have been documented, it is important to shed light on additional therapies, filling up the lack of response to first-line treatments [12,13,14]. However, steroid-resistant n-irAEs represent a poorly characterized category of patients, with a few therapeutic indications present in the recent literature. Furthermore, given the diversity of ICIs neurological toxicities and their wide range of clinical spectrum and outcomes, it is unlikely that all neurotoxicity should be treated similarly. Up to now, the efficacy of high-dose GCs in most patients has been widely confirmed [5,10,15,16,17,18]. On the other hand, the use of additional immunomodulators (e.g., including infliximab, natalizumab, MPM and cyclosporine) has been also described, obtaining good outcomes [9,18,19,20]. Moreover, cytokine blocking (e.g., JAK and IL-6) may be helpful for certain patients whose n-irAEs are severe and when steroid unresponsive [21,22].

Herein, we aim to provide a comprehensive overview and update of acute and chronic treatments of high-grade n-irAEs, focusing on steroid-refractory clinical forms. Moreover, we highlight discrepancies between current guidelines and real-life clinical setting management.

### Objectives

The primary aim was to provide an updated illustration of the most reported and best-characterized treatments of high-grade n-irAEs. The secondary aim was to give an overview of clinical characteristics, relative management, and outcomes of steroid-resistant high-grade n-irAEs. Third, we provided current algorithms and therapeutic strategies adopted in the recent literature and we analysed discrepancies between current guidelines and real clinical setting management.

## 2. Materials and Methods

### 2.1. Search Strategy

We comprehensively searched PubMed, Scopus, Embase, and Google Scholar until 31 January 2024. The following search string was used: (“neurology” OR “neurologic complications” OR “immune-related adverse events”) AND (“immune checkpoint inhibitors” OR “anti-CTLA-4” OR “anti-PD-1” OR “anti-PD-L1” OR “ipilimumab” OR “tremelimumab” OR “nivolumab” OR “pembrolizumab” OR “atezolizumab” OR “avelumab” OR “durvalumab” OR “cemiplimab”) AND (“treatment” OR “treatments” OR “corticoresistant” OR “steroid-resistant” OR “steroid unresponsiveness”). The literature search was limited to studies written in the English language. Titles and abstracts were screened to filter the results. By looking over each study’s reference list, further articles were found and screened. Based on the authors’ declarations to exclude overlapping cases, every study was carefully examined to see if the extracted cases had been previously reported in other papers. An electronic database that the authors shared was used to help with this phase. After determining which studies were pertinent, data were separately taken from each article.

### 2.2. Inclusion and Exclusion Criteria

The included studies address general and steroid-resistant high-grade n-irAEs in individuals treated with ICIs. The following clinical data needed to be present at the study level for it to be considered for inclusion: (I) type of tumour; (II) ICI treatment; (III) high-grade n-irAEs presentation, defined as CTCAE ≥ II and clinical phenotype compatible with SNC/PNS involvement after ICI administration; (IV) the course of treatment; and (V) the outcome. Studies or clinical trials without this collection of data were not selected. Furthermore, studies that looked at patients whose neurologic symptoms started before ICI treatment were not considered in the qualitative analysis. A predefined definition of outcome was not given because of the anticipated variations in follow-up times and clinical descriptions. On the other hand, the outcome was deduced based on the clinical observation at the most recent follow-up reported. For classifying deterioration or death as a result of tumour development, only the neurologic involvement was taken into account. Other comorbidities were not considered. Hence, the type of n-irAE, severity of irAE, the onset of irAE after initiation of ICI therapy, the first-line and second-line management, the time to first response, and the clinical outcome data were collected after inclusion and exclusion criteria application. Management recommendations for the treatment of n-irAEs from the American Society of Clinical Oncology (ASCO), National Comprehensive Cancer Network (NCCN), and European Society for Medical Oncology (ESMO) guidelines were compared to real-life clinical treatments from different studies.

### 2.3. Statistical Analysis

The frequency and courses of high-grade n-irAEs were summarized using descriptive statistics. A quantitative analysis of the total data is not applicable due to a higher percentage of aggregated data and missing clinical pieces of information.

## 3. Available Treatments and Possible Biological Mechanisms of High-Grade and Steroid-Resistant n-irAEs

**Figure 1:**
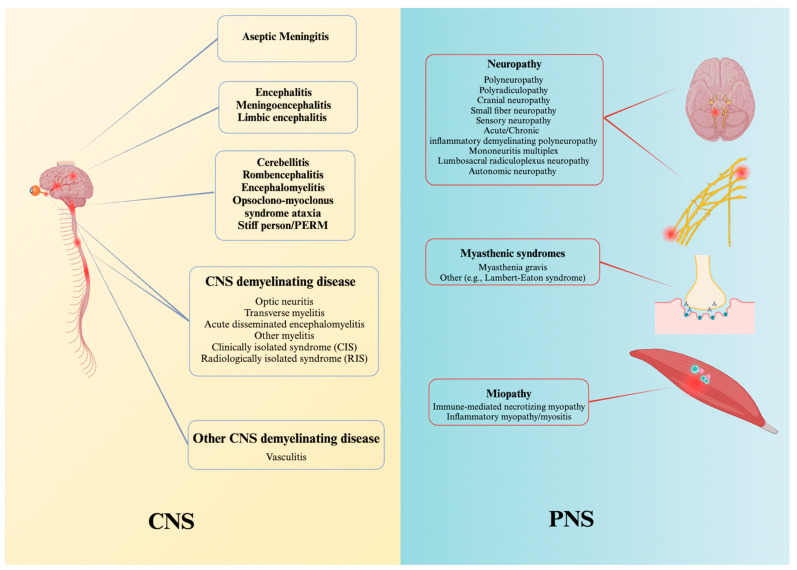
The spectrum of possible autoimmune manifestations involving the CNS and PNS according to consensus disease definitions for n-irAEs of ICIs [13]. Abbreviations: PERM, Progressive Encephalomyelitis with Rigidity and Myoclonus.

**Figure 2:**
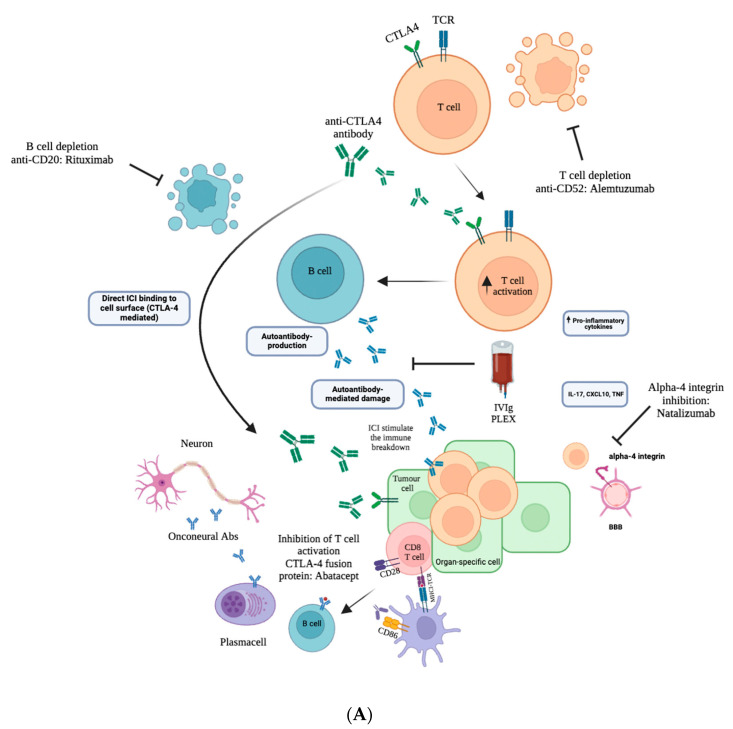

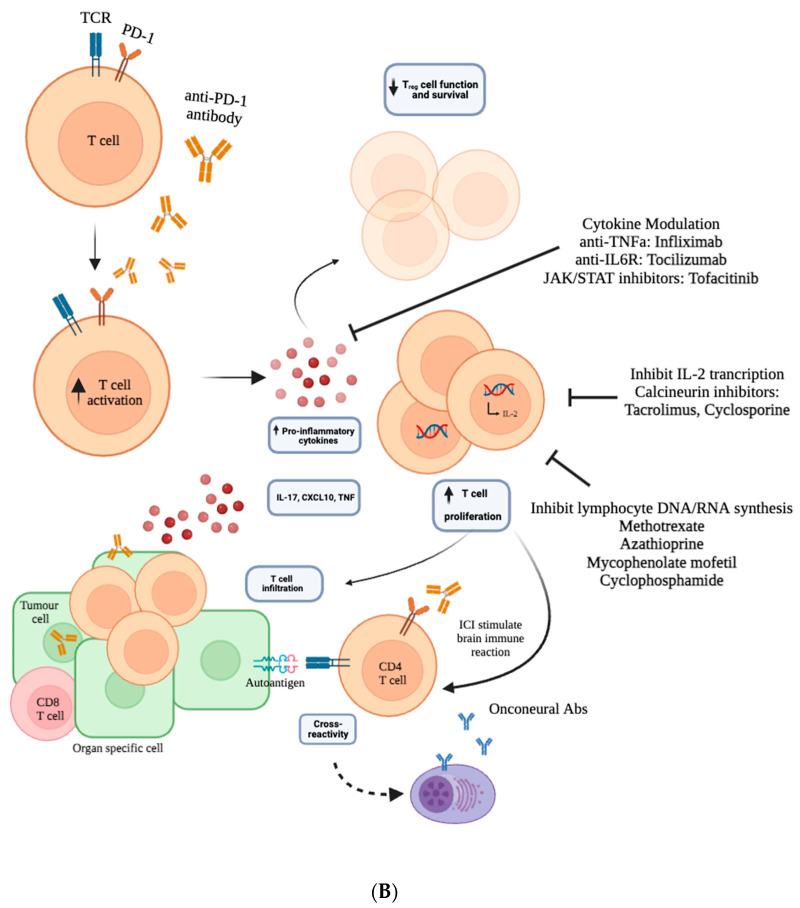
(**A**) Possible mechanisms of pathogenesis of steroid-responsive/steroid resistant PNS/CNS-irAEs and therapeutic proposals. CTLA-4 inhibitors can induce T cell activation and proliferation and Treg/TH_17_ cells impairment, in addition to autoantibody production and the induction of cross-reactivity between anti-tumour T cells and antigens on healthy cells. Biological pathways represented in the panel could be blocked by anti-CD52, anti-CD20, alpha-4 integrin inhibitors, IVIg, and PLEX. (**B**) Possible mechanisms of pathogenesis of steroid-responsive/steroid resistant PNS/CNS-irAEs and therapeutic proposals. PD-1 and PD-L1 inhibitors lead to a reduction in Treg cell survival and Treg cell inhibitory function and an increase in cytokine production and T-cell proliferation leading to brain-immune reaction. Biological pathways represented in the panel could be blocked by anti-TNFa, anti-IL6R, JAK/STAT inhibitors, IL-2 inhibitors, calcineurin inhibitors and other immunosuppressants that inhibit DNA/RNA synthesis.

We provided a schematic summary of the best available treatments for high-grade n-irAEs (Table 1) and inclusively depicted the broad clinical spectrum existing in the literature (Figure 1). We also represented possible pathogenetic mechanisms and different biological pathways of principal pharmacological targets (Figure 2A,B). The removal of self-tolerance seems to be the trigger of immunotherapy toxicities, but the precise mechanism and their chronology are unclear. Specifically, the mechanisms of immune-related adverse events owing to ICIs depend on the type of ICI used (anti-PD-1 or anti-PD-L1 inhibitors versus anti-CTLA-4 inhibitors) [23,24,25]. CTLA-4 inhibitors can induce several cellular alterations, such as T cell activation and proliferation, impaired regulatory T cell (Treg cell) survival and increased counts of type 17 T helper cells, in addition to the induction of cross-reactivity between anti-tumour T cells and antigens on healthy cells and autoantibody production (Figure 2A) [24,25]. PD-1 and PD-L1 inhibitors lead to a reduction in Treg cell survival and Treg cell inhibitory function and an increase in cytokine production. The removal of self-tolerance seems to be the trigger of immunotherapy toxicities, but the precise mechanism and their chronology are unclear (Figure 2B) [23,25]. The shift towards pro-inflammatory profile of T lymphocytes dominated by Th1/Th17 differentiation with an increased production of pro-inflammatory cytokines will promote a self-reactivity mediated either by autoreactive antibody production and/or activation of potentially self-reactive T cells. Additionally, the similarity between certain normal tissue antigens and tumour neo-antigens leads to a phenomenon of cross-reactivity favouring a self-directed immune response (Figure 2A,B) [23,24,25]. Furthermore, other mechanisms are thought to be involved in the development of n-irAEs, as genetic predisposition and infectious agents, although their role remains to be well established [10]. The CTLA-4 blockade seems to increase the availability of ligands for CD28 permitting the activation of potentially self-reactive T cells [24]. This hypothesis leads to a series of therapeutic proposals in severe steroid-resistant irAEs [25].

## 4. High-Grade n-irAEs

### 4.1. High-Grade n-irAEs: Clinical Phenotypes, Treatments and Outcomes

According to a recent review conducted by Farina and colleagues [4] and as a result of the broad clinical spectrum existing in the literature, we could categorize almost eight different n-irAEs clinical syndromes: (I) encephalitis, (II) meningitis, (III) CNS demyelinating disorders, (IV) cranial neuropathies, (V) myelitis, (VI) peripheral neuropathies, (VII) myasthenic syndromes, and (VIII) myositis (Figure 1). Patients presenting with optic neuritis or acute disseminating encephalomyelitis or multiple sclerosis or neuromyelitis optica spectrum disorder (NMOSD)-like phenotypes were recently included in the group of CNS demyelinating diseases [4,13,26]. Nervous system toxicities can manifest as early as 1 to 68 weeks, with a median onset time of 4 weeks [5,27,28,40,41,42,43,44,45,46,47]. In the last 4–5 years, numerous correlations between the neurological phenotype and the type of cancer or ICIs have been identified and documented [4,5,7,26,29]. For instance, peripheral neuropathies associated with melanoma have been described more frequently, except for sensory neuronopathy, which is usually observed in individuals with neuroendocrine tumours [5,30]. Regarding ICI treatments, myositis and myasthenia should be considered as part of the same post-ICI toxicity spectrum rather than two overlapping phenotypes frequently associated with the administration of PD(L)-1 inhibitors [7]. Conversely, peripheral neuropathy has been associated with CTLA-4 inhibitor therapy (either alone or in conjunction with PDL-1 inhibitors) [7], and cranial neuropathy to CTLA-4 and PDL-1 inhibitors [29]. Hence, different pathogenic treatments should be proposed using a mechanistic approach and based on known and validated biomarkers.

#### 4.1.1. Myositis

A recent comprehensive study [5] found that between 70 and 80% of the patients demonstrated some degree of improvement after steroid treatment (about 50% of cases) either alone or in association with other treatments (about 40% of cases). About 10% of the patients had their symptoms stabilized, while 17% of them passed away from myositis, respiratory failure, or unexpected cardiac arrest [4,5,15,31,32,33,34,48]. Relapses in symptoms were documented in a small number of survivors. Recently, in an observational study [48], three cases of myositis presenting with respiratory failure and initially misdiagnosed as myasthenia gravis have been described; two of them recovered completely after high-dose steroids.

#### 4.1.2. Myasthenic Syndromes

The majority of reported patients from aggregated data (70%) showed a positive response to therapy with steroids and steroids plus second-line medications, whereas relapse cases were rare (4%) [5,33]. However, the death rate from ICI-induced MG was substantial (about 28%), with respiratory failure accounting for 56% of reported deaths [5,7,35,36,37]. Despite receiving corticosteroid treatment, the patient with LEMS described in other studies continued to deteriorate and tested positive for Abs against P/Q-type voltage-gated calcium channels [5,38,49]. ICI-induced MG can be steroid-resistant, thus requiring second- (e.g., PLEX, IVIG) or even third-line (e.g., Rituximab) treatments in up to 30% of patients [50].

#### 4.1.3. GBS and Other Peripheral Neuropathies

Most Guillain–Barre-like syndrome patients who received steroids, either alone or in combination with other treatments, reported improvement (about 90%) [5]. The majority of patients (about 80%) reported either a partial or full recovery, 12 (13%) reported no significant reaction, and 10 (11%), died. Crucially, relapses were noted following ICI doses (15/94, 16%) or the cessation of neurologic therapy [5].

#### 4.1.4. Cranial Neuropathies

Often individuals receiving treatment with a combination of multiple ICIs develop cranial neuropathy [5,29]. From the recent systematic review [5], it was found that about 13% of patients who did not exhibit any improvement had optic neuropathy and cochleovestibular neuropathy. The vast majority of the patients (about 90%) had a full recovery or a partial improvement after steroid administration, either alone (60–70%) or in association with second-line treatments (20–30%). Moreover, about 10% of cases had relapses, which typically happened after decreasing corticosteroid doses.

#### 4.1.5. Encephalitis

In terms of the outcomes, a partial or complete neurologic improvement was seen in most cases after steroids or steroids plus other treatments (about 70%) and just 4% of cases experienced a relapse [5]. Due to the neurologic condition or its complications, about 21% of patients died. On the other hand, about 11% did not exhibit any amelioration of symptoms [5]. Up to 40% of ICI-induced encephalitis reported in the literature appear to be steroid-resistant and carry a worse prognosis than other CNS-irAEs despite second- and third-line treatments [4,41,42,43,51,52,53]. Moreover, the majority of ICI-induced encephalitis are related to Nivolumab, alone or in combination [54].

#### 4.1.6. Meningitis

The recovery in this clinical phenotype is excellent. About 15% showed partial improvement, and 85% fully recovered after steroids treatment (about 70% of cases) either alone or in association with other treatments (about 15% of cases) [55]. A recurrence occurred in about 8% of cases [5].

#### 4.1.7. Myelitis

Marini and colleagues reported that in the majority of cases, there was either a partial or significant improvement (about 71%) [5]. After receiving infliximab, a patient who had not responded to IV corticosteroids, PLEX, or cyclophosphamide had a notable improvement. About 29% of patients experienced a relapse. These findings have been confirmed by a recent case series [56]; thus, intensive immunomodulation often requiring upfront IVIg + IVMP is suggested for all patients presenting with ICI-induced transverse myelitis.

#### 4.1.8. CNS Demyelinating Disorders

The entire spectrum of demyelinating disorders of the CNS has been described in patients treated with ICI, including optic neuritis (mainly associated with ipilimumab [57]), AQP4 Abs positive NMOSD [58], ADEM [59], and Clinically Isolated Syndrome (CIS) [23]. A recent systematic review demonstrated that following the cessation of ICI and neurologic therapy with steroids either alone (25%) or in conjunction with other medications (75%), the clinical course was often monophasic, with no relapses [5]. Only 12% of patients presented a relapse [5].

## 5. Real-Life Clinical Setting versus Guidelines: A Critical Point of View

In the last decades, several research groups have focused on treatment guidelines to propose their management algorithms for treating high-grade n-irAEs. A recent expert consensus committee has published guidelines for the management of n-irAEs [44]. Up to now, the management of various clinical symptoms associated with n-irAEs is not supported by strong scientific data. Hence, there are no currently validated strategies for the management of high-grade n-irAEs in a real-life clinical setting [23,49]. 

In the case of CTCAE = II n-irAEs, the current guidelines rely on the early diagnosis of toxicities and the prompt beginning of treatment with steroids [35]. If no improvement is reached (CTCAE > II), a higher dose of steroids is initially recommended, although steroids tapering and other immunosuppressive treatments are required in case of worsening or atypical clinical presentation [35]. Overall, the first strategy involves stopping ICIs as soon as a n-irAE is suspected and using steroids (orally or IV). Subsequently, in CTCAE III/IV cases, immunosuppressive treatments (IVIg or PLEX) should be utilized in case of partial response or unresponsiveness to steroids [6,13,50,51,52].

Although guidelines are helpful resources for physicians, it is also noteworthy to remember that the CTCAE grading system is essentially a nomenclature to stratify the clinical phenotype severity. Therefore, n-irAEs can have arbitrary and ambiguous initial grading that is not always indicative of their potential severity. In fact, in clinical practice, the scenario is increasingly complex and jeopardizes the stability of the guidelines, especially in the context of steroid-resistant cases. In a real clinical setting, an escalation therapy is not always preferred, as there are reported cases in which concomitant agents that selectively interrupt the IL-6 pathway have been beneficial (e.g., Tocilizumab). Moreover, in addition to corticosteroids, IVIg or PLEX are reported to be effective as first-line therapy in CTCAE III/IV n-irAEs, suggesting that it should be considered also in steroid-refractory cases. Accordingly, different treatment algorithms have recently been proposed with some personal management suggestions [4,5,9,20,26,53]. Generally, what emerges from a real-life clinical setting is that treatment approaches against high-grade n-irAEs are heterogeneous and they are based on individual cases alone. Specifically, Reynolds and Guidon suggested that IVIg or PLEX may be used for patients with severe disease at onset or who are refractory to steroids alone [41]. Some patients will need a steroid-sparing agent if they are unable to wean off of steroids without symptom recurrence. Because there are no data to guide the selection of this treatment, neurologists and oncologists should typically consider this on a case-by-case basis. In line with recent guidelines, Anderson and colleagues previously suggested that the treatment approach must be individualized according to specific clinical phenotype, favouring pulsed steroids as first-line treatment (GC 1 g IV × 5 days) in most cases [34]. As previously reported, if symptoms do not improve with steroids alone, other immunosuppressants are needed. However, in these severe cases, in a real-life clinical setting, the same evidence considered IVIg or PLEX as a sort of add-on treatment [29,34]. According to these suggestions, myositis, MG phenotype and acute polyradiculoneuropathy, which carry a worse prognosis, should be treated with IVIg (2 g/kg over 5 days) or PLEX (at least three–five sessions) in association with corticosteroids. In contrast to the escalation therapy approach reported in the guidelines, clinical practice reveals that IVIg or PLEX should be recommended in steroid-refractory cases as first-line treatment besides corticosteroids in CTCAE III/IV cases of GBS, MG and myelitis [20]. Therefore, according to the literature findings and our experience, we suggest that it could be possible to distinguish the treatment of CNS n-irAEs from PNS once (Table 2). It would be necessary to tailor the treatment of these patients based on their specific clinical phenotype, which varies from patient to patient, despite the same clinical syndrome, a condition not fully considered by the current guidelines. Accordingly, here we present the qualitative results and our clinical experience, taking into account a clinical context that varies from patient to patient and centre to centre, which is less standardizable compared to the guidelines. Given the clinical phenomenological complexity of these patients, we suggest that the CTCAE scale is not entirely comprehensive for selecting the first-line standard treatment according to the guidelines, in both general and steroid-resistant high-grade n-irAEs. For example, independently from the guidelines, in the case of CNS n-irAEs (e.g., encephalitis), the use of broad-spectrum immunosuppressants (e.g., CPM, RTX) is beneficial, if improvement has not occurred with corticosteroids alone [4,54,55,56]. After 4–6 weeks of worsening, oral immunosuppressants (MPM, AZA, MTX) may be also taken into consideration [10,29]. 

Conversely, as reported before, PNS n-irAEs (e.g., MG, myositis or GBS phenotypes) may receive early IVIg and/or PLEX therapies that have been reported to be beneficial [50,57,58]. Generally, most of the PNS n-irAEs are corticoresponsive and a low–moderate dose of steroid is sufficient. However, despite guidelines promoting more escalation therapy depending on the CTCAE score, in some cases, a more aggressive approach is needed from the start of treatment. In particular, in a real-life clinical scenario of GBS phenotype (e.g., CTCAE III/IV cases), PLEX (five treatments over 2 weeks) or IVIg (2 g/kg over 5 days) are considered as first-line management, in combination with pulsed steroids, followed by a slow tapering in 4–6 weeks [20,28]. Considering the MG phenotype, IVIg (2 g/kg over 5 days) is required in the most severe cases (CTCAE II/III/IV) and RTX or PLEX should be added in refractory cases [34]. Regarding the irM phenotype, Bompaire and colleagues observed that in CTCAE II/III/IV cases, ICI treatment should be withheld, and IV corticosteroids should be started. Steroid-sparing agents alone might be considered in mild irM cases when hospital admission is not required [60]. Additionally, life-threatening forms (e.g., with bulbar and/or respiratory muscles involvement or concurrent myocarditis) require admission in ICU and treatment with IVIg or PLEX is needed. Furthermore, RTX may be favoured early in patients with seropositivity for myositis-specific antibodies (Table 1). Indeed, the use of ABT is supported by single case reports in which it has proved effective against the most severe forms of irM [4].

The literature evidence and our experience indicate that age, underlying malignancy, and specific clinical phenotype at presentation are the key factors influencing the likelihood of clinical improvement of n-irAE patients [4]. On the contrary, we suggest that the choice of treatment for high-grade n-irAEs, considering the CTCAE scale compared to real-world clinical data, is not sufficient to improve the outcome for each specific high-grade phenotype. However, due in part to the small size of previously reported cohorts, studies about the neurological outcomes of these varied complications are inconsistent [10,16,61]. Specifically, most of the outcome data existing in the literature appear to be aggregated data, as well as difficult to separate and analyse per single treatment. To date, Farina and colleagues investigated the largest cohort of n-irAEs (including high-grade n-irAEs and steroid-resistant cases) [4]. They reported and showed that around half of the cases resulted in neurological recovery, whereas a third of the patients died during the research period, primarily due to tumour progression and neurological damage [4]. In line with previous findings [15,49], they obtained that older age and paraneoplastic-like syndromes are linked to decreased neurological recovery. Moreover, MG/myositis related to ICI treatment and melanoma are associated with a higher probability of clinical improvement [4]. In their cohort, MG/myositis cases were treated principally with steroids, and only a minor part required other treatments (e.g., IVIg, PLEX, RUXO) [4]. Conversely, other research evidence comes from smaller cohorts [18]. For instance, Diamanti and colleagues observed that patients who belong to high-grade n-irAEs or who were considered corticoresistant, had better outcomes after IVIg or PLEX treatment [18], taking a more aggressive initial approach without waiting for an escalation therapy.

However, analysing their cohort, it is not possible to differentiate between high-grade and steroid-resistant cases, to conduct quantitative outcome analyses. It is likely that the heterogeneous response reported in the literature and according to our experience, dependent on patient phenotype and characteristics, is quite different because current guidelines still do not allow for differentiation among various patient subgroups, leading to a standard of care at the expense of sometimes lower overall efficacy.

**Table 2:** High-grade ICI-induced n-irAEs in the central and peripheral nervous systems, therapies, associated outcomes, and ICI rechallenge.

Type of irAE	Grade of Neurological Toxicity	Suspected Causing Agents	Estimated Frequency	Timing after First ICI Dose #	Immune-Modulating Treatments (First, Second, Third Line)	Reported Outcome	ICI Rechallenge	Selected Reported Cases (References)
Encephalitis	CTCAE ≥ II	Nivolumab, pembrolizumab, nivolumab + ipilimumab	0.5–13%	within 8–16 weeks	Corticosteroids IVIg Cyclophosphamide Rituximab Natalizumab	After permanently discontinuing ICI and starting a trial of steroids: partial or full improvement (68–76%); relapse (12%); stable (11%); and death (21%).	Not considered in clinical practice	[5,7,42,51,52,53,62,63,64]
Aseptic meningitis	CTCAE ≥ II	Ipilimumab	3%	within 12–16 weeks	Corticosteroids IVIg Infliximab	After withdrawal of ICI and steroid therapy: usually complete recovery (90–100% of cases).	If grade ≤ 2 and complete recovery	[5,9,55,65]
Myelitis	CTCAE ≥ II	Nivolumab, pembrolizumab, nivolumab + ipilimumab	2%	within 12–16 weeks	CorticosteroidsIVIg PLEX Cyclophosphamide, Infliximab Tocilizumab	Partial or full improvement (71%) and stable (29%).	NA	[5,16,21]
CNS demyelinating diseases	CTCAE ≥ II	Nivolumab, pembrolizumab, nivolumab + ipilimumab	6%	NA	Corticosteroids Corticosteroids + (IVIg PLEX Methotrexate Infliximab, Rituximab)	Partial or full improvement (75%), stable (12%) and death (12%).	It is possible if grade ≤ 2 and complete recovery	[4,5,29]
Acute immune demyelinating Polyneuropathy	CTCAE ≥ II/III	Nivolumab, pembrolizumab, ipilimumab	0.1–7.6%	within 12–16 weeks	Corticosteroids IVIg PLEX Tacrolimus Infliximab Rituximab	Partial or full improvement (77%), stable (13%), and death (11%).	NA	[5,7,10,17,33,51,53,66]
Chronic immune demyelinating Polyneuropathy		Nivolumab, pembrolizumab, nivolumab + ipilimumab		within 12–16 weeks	Corticosteroids IVIg PLEX Mycophenolate mophetil Infliximab Rituximab		NA	[5,53]
Neuropathy (GBS + other neuropathy)	CTCAE ≥ II/III	Nivolumab, pembrolizumab, ipilimumab	Up to 22%	within 12–16 weeks	Corticosteroids IVIg PLEX Mycophenolate mophetil Tacrolimus Infliximab Rituximab	Steroids Steroids + others (IVIg, PLEX, infliximab, tacrolimus, mycophenolate, and rituximab) IVIg PLEX	Attitude toward rechallenge is generally reluctant, however it is possible if grade ≤ 2 and complete recovery	[5,7,10,17,33,42,43,51,53,66,67,68]
Cranial nerves neuropathies	CTCAE ≥ II	Pembrolizumab, ipilimumab	7%	within 12–16 weeks	Corticosteroids Steroids + others (IVIg, PLEX, Mycophenolate mophetil, Infliximab, Rituximab)	Partial or full improvement (87%) and stable (13%).	If grade ≤ 2 and complete recovery	[5,10,42]
Myopathy	CTCAE ≥ II	Ipilimumab, nivolumab, pembrolizumab, nivolumab + ipilimumab	Up to 32%	Within 2–8 weeks	Corticosteroids IVIg PLEX Mycophenolate mophetil Tocilizumab Infliximab JAK-inhibitors	After temporary withdrawal of ICI and permanent discontinuation in patients with myocardial involvement: partial or full improvement (75–84%), stable (10%) and death (15–20%; up to 50% with concomitant myocarditis).	If grade < 1 and complete recovery	[5,15,23,47,53,61,69,70,71]
Myasthenic syndromes	CTCAE ≥ II/III	Ipilimumab, nivolumab, pembrolizumab, nivolumab + ipilimumab	Up to 14%	Within 4–24 weeks	Corticosteroids IVIg PLEX Cyclophosphamide, Mycophenolate mophetil Rituximab Ruxolitinib	After withdrawal of ICI and steroid starting: partial or full improvement (70%), stable (3%) and death (28–30%).	If grade = 2 and complete recovery	[5,7,44,53,72,73,74]

# delayed presentations possible.

### Proposed Treatment Algorithms for Steroid-Resistant High-Grade PNS/CNS-irAEs: The Literature Findings and Our Experience

After a careful analysis of the literature, a few poorly characterized cases of steroid-resistant n-irAEs were found, and only aggregated data were reported (Table 3). Therefore, it is worth mentioning that more systematic studies on the inadequately standardized therapy of steroid-refractory patients are needed. However, immunomodulatory or immunosuppressive approaches such as IVIg, PLEX, TNF-α antagonists, and AZA or MPM were already reported to be beneficial in the recent literature [5,18,19,33,75]. Rapid escalation to IVIg, CPM, or RTX is also advocated [16,35]. It was also recently documented that performing promptly PLEX may accelerate ICIs clearance and it has been proposed in all cases with high-grade steroid-resistant n-irAEs, even though its optimal schedule in this context is unknown [75].

Surprisingly, after the literature analysis, it is noteworthy to observe that the estimated frequency of high-grade steroid-resistant n-irAEs should not be underestimated, considering the high rate of severe outcomes (Table 3). Although data are very heterogeneous, overall, a frequency of clinical phenotype that is distinct from other high-grade n-irAEs emerges, as minimally mentioned in previous studies [18,60]. Particularly, the clinical phenotypes with higher steroid resistance rates are myelitis, cranial neuropathies, GBS/other neuropathies, and CNS demyelinating disorders, followed by aseptic meningitis, encephalitis, myopathies and neuromuscular disorders (Table 3). Additionally, we found that Nivolumab, Pembrolizumab and Ipilumab are the most associated ICI related to steroid-resistant PNS/CNS n-irAEs (Table 3).

**Figure 3:**
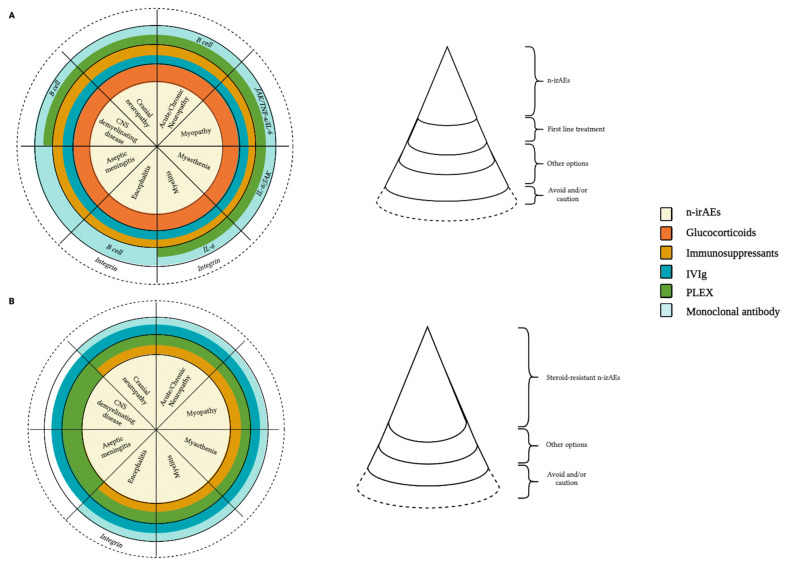
Suggested therapeutic algorithm for the management of steroid-sensitive (**A**) and steroid-resistant n-irAEs (**B**). When a systemic therapy is considered in patients presenting with steroid-resistant n-irAEs owing to immune checkpoint inhibitors, therapies to be considered depend on the affected organ system but can include immunosuppressants, IVIg, PLEX and monoclonal antibodies. Treatments to avoid or to use with caution are also reported according to different phenotypes, although discrepancies on the real effectiveness of Natalizumab for encephalitis-irAE treatment were found. These therapeutic suggestions are based on recommendations included in official guidelines, data from some retrospective studies, isolated published cases and personal experiences of the authors.

Moreover, from our qualitative analysis emerged that the management of steroid-resistant PNS/CNS-irAEs is poorly standardized [28,47,49,50,69,73,75,76,77]. For instance, after one week of high-dosage steroid therapy, if PNS-irAEs do not improve, the immunosuppression strategy needs to be increased right away. In these cases, IVIg and PLEX are the most often used second-line therapy. TCZ, Infliximab, RTX, MPM, MTX, and CPM are the most often adopted alternative treatment strategies [9,61,78]. In rare instances, other molecules have been suggested, such as calcineurin inhibitors (e.g., Tacrolimus, Cyclosporine), proteasome inhibitors (e.g., Bortezomib), IL-17 blockers, and T-cell depleting monoclonal antibodies (anti-thymocyte globulin) [61,78]. Interestingly, ABT (immunoglobulin IgG1 Fc region linked to the extracellular domain of CTLA-4; Figure 2A,B) is effective in treating abrupt exacerbation of preexisting myositis that is not responsive to high-dose steroids [70]. Despite the promising data on a small number of patients, these agents are still being used empirically. Specifically, we reported a collective analysis from the literature search and comprehensive of our experience, suggested therapeutic algorithms for both high-grade and steroid-resistant n-irAEs, as shown in Figure 3.

Accordingly, the management of high-grade cortico-resistant CNS-irAEs patients is not well defined, and the treatment options should be chosen after weighing the potential advantages and disadvantages of each one. Recent evidence reported that immunosuppressants (e.g., CPM and RTX) should be taken into consideration in patients who test positive for high-risk antibodies, have severe presentations (e.g., loss of consciousness, severe motor weakness, refractory seizures, or massive memory deficits), or do not improve after 10 to 15 days of corticosteroid therapy [76]. Interestingly, TCZ has been successfully used in combination with JAK-inhibitor therapy in steroids-refractory myelitis [8]. Moreover, NTZ (anti-integrin that inhibits lymphocyte entry into the brain parenchyma; Figure 2A), could be another useful strategy [79]. NTZ has been reported to be beneficial in a single case of steroid-refractory limbic encephalitis [79]. Additionally, repeated PLEX resulted in clinical improvement and remission of CSF abnormalities in a case of steroid-refractory Atezolizumab and Bevacizumab-induced encephalitis [80].

On the other hand, no less important than the type of treatment is what neurologists need to know before starting treatment. We suggest that it is crucial to perform a routine neurologic history, assessment of systems, and exam before starting immunotherapy in n-irAEs patients. Any tumour or treatment-related impairments are highlighted at baseline by the neurologic exam. When deciding whether to start therapy, a history of immune-mediated neurologic diseases is particularly pertinent [44]. But it is also critical to be mindful of other illnesses, such as migraine or hereditary muscular disease, as these may exacerbate baseline neurologic dysfunction or make it more difficult to diagnose new symptoms that may indicate an n-irAE. Pre-ICI neurologic and laboratory evaluation guidelines are constantly changing [44]. A range of pretreatment laboratory analyses are taken into account in the recommendations made by the Society for Immunotherapy of Cancer. However, from a neurologic standpoint, routine blood counts and chemistries are not as important as baseline CK, thyroid stimulating hormone, and free thyroxine values [6,44]. ICI therapy is presently started or continued on a case-by-case basis if a patient has a suspected or confirmed neurologic disease. Maximizing oncological treatment options for patients while reducing potential morbidity and mortality associated with n-irAEs is the aim of coordinated and interdisciplinary care [44].

However, there is currently insufficient data to support or contradict these strategies in the case of high-grade PNS/CNS-irAEs [76]. Further investigation into the underlying immunopathogenesis is necessary to create treatment plans mostly appropriate for high-grade PNS/CNS-irAE and in particular for steroid-resistant clinical forms.

**Table 3:** High-grade n-irAEs unresponsiveness (including relapses and excluding deaths) to GCs (oral/IV or both) within the first 7–14 days of associated therapies and outcomes.

Steroid-Resistant n-irAEs	Grade of Neurological Toxicity	Suspected Causing Agents	Estimated Frequency of Steroid-Resistant Cases	Immune-Modulating Treatments (Second, Third Line)	Reported Outcome or CTCAE	Selected Reported Cases (References)
Encephalitis	CTCAE II/III/IV	Nivolumab, pembrolizumab, nivolumab + ipilimumab	3–4%	IVIg (0.4–2 g/kg/day cumulative dose within 3–5 days) PLEX (5–7 sessions) MMF Rituximab Natalizumab	Death (CTCAE V)	[5,18,26,44,53,79,80,81,82,83]
Aseptic meningitis	CTCAE II/III/IV	Ipilimumab	Up to 8%	IVIg (2 g/kg/day cumulative dose within 3–5 days) PLEX (5–7 sessions)	NA	[5,26,44]
Myelitis	CTCAE II/III/IV	Nivolumab, pembrolizumab, nivolumab + ipilimumab	Up to 14–40%	IVIg (2 g/kg/day cumulative dose within 3–5 days) PLEX (5–7 sessions) Tocilizumab + JAK inhibitor	NA	[5,11,16,21,26,33,44,81]
CNS demyelinating diseases	CTCAE II/III/IV	Nivolumab, pembrolizumab, nivolumab + ipilimumab	Up to 4–13%	IVIg (1 g/kg/day cumulative dose within 3–5 days) PLEX (5–7 sessions)	Stable deficit (CTCAE II)	[4,5,18,42,81,84]
Acute immune demyelinatingPolyneuropathy	CTCAE II/III/IV	Nivolumab, pembrolizumab, ipilimumab	Up to 13–17%	IVIg (2 g/kg/day cumulative dose within 3–5 days) PLEX (5–7 sessions) Tacrolimus Rituximab	CTCAE IV	[18,33,36,53,66,85]
Chronic immune demyelinatingPolyneuropathy	CTCAE II/III/IV	Nivolumab, pembrolizumab, nivolumab + ipilimumab		IVIg (2 g/kg/day cumulative dose within 3–5 days) PLEX (5–7 sessions)	CTCAE IV	
Cranial nerves neuropathies	CTCAE II/III/IV	Pembrolizumab, ipilimumab	Up to 23%	IVIg, PLEX, Infliximab, MPM, and Rituximab	NA	[5,29]
Myopathy	CTCAE II/III/IV	Ipilimumab, nivolumab, pembrolizumab, nivolumab + ipilimumab	Up to 2–8%	PLEX (5–7 sessions) and IVIg (0.4–2 g/kg/day cumulative dose within 3–5 days) Infliximab Bortezomib Tacrolimus Abatacept Rituximab	From partial remission to death (CTCAE III-V)	[5,15,18,23,26,36,44,53,73,81,85,86,87,88,89,90]
Myasthenic syndromes	CTCAE II/III/IV	Nivolumab Pembrolizumab Atezolizumab	Up to 5%	PLEX (5–7 sessions) and IVIg (0.4–2 g/kg/day cumulative dose within 3–5 days) Azathioprine, Cyclosporine, MPM RTX TCZ ABT	NA	[5,33,36,53,88]

## 6. Rechallenging with ICI

Rechallenge with an ICI is discussed on a case-by-case basis depending on the oncological and neurological evolution [45,60]. Regarding the reintroduction of immunotherapy in patients with PNS-irAEs (e.g., irM, MG phenotype), there is no clear consensus, although some recommendations are reported in the recent literature (Table 2). In a shared decision-making process, the choice to retry ICI should be based on criteria about the tumour (response to ICI, alternative therapeutic choices, and prognosis) as well as the irAE severity (extent of necessary immunosuppressive regimen) [26,41,91]. As long as the therapeutic response to immunosuppressive treatment has been sufficient, the recent literature proposes that rechallenging could be tried in a limited number of patients with mild-to-moderate or even moderate–severe n-irAEs (e.g., irM). It is not advised for patients who have bulbar or respiratory symptoms and an MG phenotype, associated or not with myocarditis. On the other hand, current guidelines recommend discontinuing ICI treatment permanently when high-grade CNS-irAEs occur (e.g., demyelinating disease with transverse myelitis, leukoencephalopathy or PRES) [6,35]. No specific recommendations are given for encephalitis and aseptic meningitis. Therefore, the evidence guiding the choice to safely reintroduce ICIs in patients with CNS-irAEs is scarce and mainly derived from retrospective studies. Furthermore, patients with evidence of neurologic autoimmunity before the beginning of ICI therapy, mainly in the form of PNS affecting the CNS, tend to have irreversible neurologic deterioration following ICI initiation [16]. In such patients, ICI re-challenge should be considered cautiously. In the case of meningitis, favourable neurological and oncological outcomes can be achieved after the reintroduction of ICIs [92]. Evidence supporting ICI rechallenge in patients with immune-related encephalitis is still inconclusive. However, in small cohorts of patients re-challenged with ICIs, no relapses occurred [93].

Hence, in light of the highly heterogeneous data in the literature, we firmly believe that the safety of ICI rechallenges in patients who achieve neurological recovery, both in general and steroid-resistant high-grade n-irAEs, should be the subject of further future investigations.

## 7. Trials Ongoing and Future Research

Consensual recommendations or prospective randomized controlled trials are still lacking for the treatment of n-irAEs and in particular for MG, myocarditis, and irM. Therefore, observational studies are the only sources of treatment-related evidence. The American Society of Clinical Oncologists (ASCO), the European Society for Medical Oncology (ESMO), the National Comprehensive Cancer Network (NCCN), the Society for Immunotherapy of Cancer (SITC), and the European League Against Rheumatism (EULAR) are among the organizations that have released guidelines that are based on the consensus of experts [27,91,94].

Here we report the most recent clinical trials ongoing. However, there are currently no specific clinical trials for n-irAEs. Hence, we have reported potential trials that may be tested specifically in this category of irAEs in the future.

For example, an ongoing clinical trial aims to answer whether RTX or TCZ are effective in treating irAE in patients requiring prolonged GC (NCT04375228). Moreover, there are two ongoing clinical trials to assess ABT use in immune-related myocarditis associated to n-irAEs (NCT05335928 and NCT05195645) [71]. Other treatments proposed are complement inhibitors (eculizumab, zilucoplan), B-cell activating factor (belimumab), interferon-alfa (sifalimumab), cannabinoid receptors (lenabasum), and toll-like receptors 7–8 and 9 (IMO-8400), without enough evidence to further explain in our study [31].

Furthermore, some anectodical evidence supports the role of ECP (extracorporeal photopheresis) in the treatment of ICI-induced colitis [95]; however, data from large prospective randomized trial are lacking; to date, a clinical phase 1/2 trial (DRKS00025078) is currently ongoing to determine safety and efficacy of ECP in patients with irAEs as well as its effect on tumour progression. Additionally, finding predictive biomarkers of toxicity may benefit those who are at risk for the development of n-irAEs, sparing them unnecessary pain and dictating alternative antitumour treatment strategies. There are currently some ongoing clinical trials (NCT05813418) aimed at measuring variations from baseline of certain cytokines (e.g., IL6, IL10) during ICI treatment that might relate to the risk of developing irAEs.

## 8. Discussion

In this practical review, we discussed the clinical spectrum, management, and outcome of ICI neurologic toxicities, with a focus on cortico-resistant high-grade n-irAEs. As n-irAEs constitute a diverse collection of neurological illnesses with unique properties from their idiopathic counterparts, there are continuous efforts to characterize them [4,5,76]. The severe and sometimes fatal nature of n-irAEs should not be underestimated despite their rarity [4,5,76]. Hence, to lower the chance of mortality and poor outcome of PNS/CNS-irAEs, it is crucial to think about prompt treatment as soon as possible. It is doubtful that all neurotoxicity should be not treated in the same way due to the variety of n-irAEs and their wide range of clinical phenotypes and outcomes [4,5,46]. Treatment guidelines for n-irAEs have been recently developed [13]. However, these recommendations are not even supported by a real-life setting, on which some researchers contrast various treatment modalities [53]. Bringing all together the most recent literature data, we tried to gain greater clarity on the phenotype–treatment relationship in accordance with recent literature data, although clear limitations are still evident. 

Overall, considering high-grade PNS-irAEs, 70–80% of the patients with n-irM demonstrated clinical improvement after either steroids-only therapy or in association with other treatments [5], in line with more recent evidence [4,76]. These results demonstrate the non-superiority of second-line treatments compared to single steroid treatment, although double-blind clinical trials are needed. However, from the literature search and real clinical-life setting, we found that IVIg and other immunosuppressants (e.g., Tacrolimus, CPM, MTX, MPM) are the most effective treatment in case of partial response to GCs. In MG phenotypes, we observed that the majority of patients (about 70%) showed a positive response to therapy with steroids either alone (33%) or in association (45%) [5]. It is noteworthy to consider that association with other treatments (e.g., IVIg and/or PLEX) could be beneficial. Furthermore, in patients with Lambert–Eaton myasthenic syndrome (LEMS), it was observed that additional treatments in association with steroids should be necessary to obtain clinical improvement [5,49]. Most GBS-like patients who received steroids, either alone (23%) or in combination with other treatments (50%), reported improvement in about 90% of cases [5]. Moreover, the association with steroids plus IVIg or MPM demonstrated high efficacy in clinical improvement compared to steroid-only treatment [5,29,96]. Conversely, cranial neuropathies treatment benefits more from single steroid administration, although sometimes the association with second-line treatments is needed (e.g., IVIg, PLEX, infliximab, MPM, and RTX [5]). 

Additionally, in the case of high-grade CNS-irAEs, we found that a partial or complete neurologic improvement was achieved in most encephalitis treated with the association of steroids plus other treatments (e.g., IVIg, RTX, CPM, NTZ) [5]. We collected the same results in aseptic meningitis cases, obtaining about 85% of fully recovered cases after steroid treatment alone and in minor part in association with other treatments (e.g., IVIg, Infliximab) [5]. Indeed, myelitis associated with ICIs showed partial or full recovery also after IVIg, PLEX and/or other immunosuppressants (e.g., CPM, TCZ) without association with steroids [5,16,21]. Strikingly, Infliximab showed a notable clinical improvement in patients with myelitis who had not responded to IV CGs, as well as PLEX or CPM. Therefore, CNS demyelinating disorders in some cases benefit more from association with steroids plus PLEX or CPM or IFNβ-1a and/or IVIg. Last but not least, we suggest also that is very important to consider a spectrum of n-irAEs poorly characterized, like PNS-irAEs in association with CNS deficits. The treatment of this clinical subgroup is more complicated and often requires prompt aggressive treatment (e.g., high-dose steroids or association therapy). However, also for these cases, more standardized clinical trials are needed. 

Going into more detail and focusing on steroid-resistant high-grade n-irAEs, our literature results are incredibly heterogeneous and poorly standardized, due to the lack of systematic collection of data. However, it is possible to distinguish different treatment strategies and outcomes for steroid-resistant PNS/CNS-irAEs, suggesting a probable different underlying pathogenesis. As previously mentioned, the management of steroid-resistant PNS-irAEs is poorly standardized [23,25,44,61,72,73,75]. Although previous studies reported in about 40% of PNS-irAEs that additional immunosuppressants are required due to corticoresistance, corticodependence (>10 mg prednisolone day), or ineffective steroid tapering [26], we observed that this estimated frequency, overall, is probably inferior to that which is reported. In steroid-refractory PNS-irAEs, IVIg and PLEX seem to be the most often used second-line therapy, particularly in cases of chronic immune demyelinating polyneuropathy [53]. Sometimes, alternative molecules are needed (e.g., TCZ, Infliximab, RTX, MPM, Tacrolimus, Cyclosporine, MTX, and CPM) [9,61,78]. On the contrary, cortico-resistant high-grade CNS-irAE treatment options are more heterogeneous than the previous one, depending on the potential advantages and disadvantages, as previously mentioned. For instance, in patients with CNS demyelinating disease who tested positive for high-risk antibodies, it was recommended to start treatment with CPM and/or RTX [76]. Regarding steroids-refractory myelitis, a successful combination between TCZ and JAK-inhibitor therapy was observed [21]. It was also reported that NTZ could be a useful strategy in limbic encephalitis [79] and meningoencephalomyelitis [64]. Unfortunately, more research into the underlying immunopathogenesis is required to develop therapeutic strategies that are most suitable for this type of PNS/CNS-irAE. Identifying genetic, epigenetic, or surrogate predictive markers of n-irAE development is expected to enable a better safety appraisal of ICI therapies in patients deemed at high risk of n-irAE development, guiding the development of preventive interventions [5]. The current review has some related limitations in its design. First, neither the exact incidence nor the prevalence of corticoresistant high-grade n-irAEs could be estimated, therefore prospective future studies are needed to precisely describe the epidemiologic characteristics of these clinical forms. Second, self-resolving toxicities may be less likely to be recorded, which might have an impact on the severity and death rate of neurologic sequelae due to publication bias. Finally, as our analysis is based on a retrospective synthesis of published studies, patient data may be absent or lacking, and we are unable to confirm the authors’ information and diagnoses.

## 9. Conclusions

High-grade n-irAEs treatment decisions should be made by a specialized multidisciplinary board that includes neurologists and oncologists, as this approach has been shown to improve the patient’s outcome in the general setting of irAEs [97]. Overall, considering steroid-resistant PNS/CNS-irAEs, the clinical benefit derived from the IVIg and PLEX therapies seems to be significant, although a proportion of patients with CNS-irAEs (e.g., encephalitis) and some PNS-irAEs (e.g., acute or chronic neuropathy and myopathy) still demonstrate a poor outcome (CTCAE IV/V), and therefore additional immunosuppressants are needed. Given that ICI treatment will be administered to a growing number of patients in the near future, appropriate treatment of cortico-resistant clinical forms is fundamental for a correct neurological clinical practice. Therefore, more prospective studies and randomized clinical trials are needed in steroid-resistant n-irAEs.

## Figures and Tables

**Table 1:** Properties of the current therapeutic options for high-grade n-irAEs and associated conditions. First- and second-line treatments do not necessarily correspond to the consecutive trial of the management. IVIg and PLEX are considered as acute phase treatment.

Class of Drug	Drug and Standard Dosage	N-irAEs and Associated Conditions	Selected Reported Cases
Glucocorticoids (GCs)	Oral PDN: mild: 0.5–1 mg/kg; mild-moderate: 1–1.5 mg/kg; moderate 1–2 mg/kg MPDN: severe irM, MG, myocarditis: IV MPDN (0.5–1 g/d × 3–5 d)	Mild-moderate irM cases MG (Oral and IV) GBS Myocarditis (IV) ICI-induced dermatomyositis (topical)	[19,26,27,28,29,30,31,32,33,34]
Immunosuppressant agents	FK (0.06–0.3 mg/kg/day) CYC (1–2 mg/kg/d and up to 5 mg/kg/day)	Moderate or GCs-unresponsive irM cases	[16]
	MPM (up to 3 g/d PO in divided doses or 0.5–1 g PO once every 12 h)	Chronic immune demyelinating polyneuropathy Severe Myositis	[5]
	AZA (up to 2–2.5 mg/kg/day)	Mild myositis, steroid-refractory (also for maintenance)	[35]
	MTX (20–25 mg/w, oral or IM), with daily folic acid supplements	Mild–moderate myositis, steroid-dependent myositis	[36]
	CPM (1–2 mg/kg/d)	Moderate-severe myositis	
Immunoglobulins (IVIg)	IVIG: 400 mg/kg/d for 5 d every 4–6 w	Myositis MG acute polyradiculoneuropathy (In association with steroids)	[29]
Plasma exchange (PLEX)	PLEX (3–5 cycles)	GBS moderate-to-severe MG	[18]
Biologics	RTX (375 mg/m^2^/w × 4 w)	Severe irM with seropositivity for myositis-specific antibodies G3-G4 MG (MGFA class III–IV) refractory to PLEX or IVIG	[35,36]
	IFX: 5 mg/kg	GBS cranial neuropathies	[5]
	TCZ (8 mg/kg every 4 w)	Steroid-refractory myositis and steroid-refractory encephalitis	[36,37]
	ABT (500 mg every 2 w × 5 doses)	Mild-moderate myositis and myocarditis	[4]
	ALE (single dose of 30 mg)	Steroid-refractory myocarditis	[38]
	TFC (5 mg PO, twice daily) RXL (25 mg PO, twice daily) BCT (4 mg PO, once daily)	Chronic inflammatory demyelinating polyneuropathy Acute transverse myelitis	[21,39]

Abbreviations: ABT, abatacept; ALE, alemtuzumab; AZA, azathioprine; BCT, baricitinib; CPM, cyclophosphamide; FK, tacrolimus; IFX, infliximab; IM, intramuscular; IVIG, intravenous immunoglobulins; MPM, mycophenolate mofetil; MPDN, methylprednisolone; MTX, methotrexate; PDN, prednisone; PLEX, plasma exchange; PO, per oral; RTX, rituximab; RXL, ruxolitinib; TCZ, tocilizumab; and TFC, tofacitinib.

## Data Availability

Not applicable.

## Abbreviations

CNS: central nervous system; PNS: peripheral nervous system; irAE: immune-related adverse event; ICIs: immune checkpoint inhibitors; PD-1: programmed cell death protein 1; PDL-1: programmed death-ligand 1; CTLA-4: cytotoxic T-lymphocyte-associated protein 4; GCs: glucocorticoids; IVIg: intravenous immunoglobulins; PLEX: plasmapheresis; AZP: azathioprine; BRZ: bortezomib; CPM: cyclophosphamide; IFX: infliximab; MPM: mycophenolate mofetil; RTX: rituximab; and TCR: tacrolimus.
